# Proteasome Inhibition by Carfilzomib Induced Apotosis and Autophagy in a T-cell Acute Lymphoblastic Leukemia Cell Line

**DOI:** 10.22037/ijpr.2020.112692.13898

**Published:** 2019

**Authors:** Maryam Sadat Hosseini, Mohammad Hossein Mohammadi, Rouhollah Vahabpour Roudsari, Leila Jafari, Pargol Mashati, Ahmad Gharehbaghian

**Affiliations:** a *Department of Laboratory Hematology and Blood Bank, School of Allied Medical Sciences, Shahid Beheshti University of Medical Sciences, Tehran, Iran. *; b *HSCT Research Center, Department of Laboratory Hematology and Blood Bank, School of Allied Medical Sciences, Shahid Beheshti University of Medical Sciences, Tehran, Iran. *; c *Department of Medical Lab Technology, School of Allied Medical Sciences, Shahid Beheshti University of Medical Sciences, Tehran, Iran.*

**Keywords:** Acute lymphoblastic leukemia, Molt4 cells, Autophagy, Apoptosis, Proteasome, Carfilzomib, Dexamethasone

## Abstract

T-cell acute lymphoblastic leukemia is an aggressive hematologic malignancy which is usually associated with unfavorable prognosis particularly in patients with refractory/relapsed disease. Therefore, development of novel therapeutic strategies is highly required for improving the outcome of these patients. Although there are several studies evaluating the efficacy of proteasome inhibitors on acute lymphoblastic leukemia of B-cell lineage, the data are still limited regarding T-cell acute lymphoblastic leukemia. Here, we tried to investigate the effects of the proteasome inhibition by carfilzomib on the induction of apoptosis and autophagy in Molt4 cells. The effect of carfilzomib in combination with dexamethasone in Molt4, as a glucocorticoid-resistant T-cell acute lymphoblastic leukemia cell line, was also assessed. Our data showed that carfilzomib can induce both apoptosis and autophagy in Molt4 cells. Furthermore, we found that carfilzomib is a potent inducer of reactive oxygen species production and also induces G2/M phase cell cycle arrest in Molt4 cells. Concomitant treatment with carfilzomib and dexamethasone demonstrated that carfilzomib can synergistically enhance the cytotoxic effect of dexamethasone on Molt4 cells. Furthermore, co-treatment of the cells with carfilzomib and dexamethasone increased the induction of autophagy as compared with each drug alone. In conclusion, our results are suggestive of the effectiveness of carfilzomib on Molt4 cells as a model of GC-resistant T-cell acute lymphoblastic leukemia.

## Introduction

T-cell acute lymphoblastic leukemia (T-ALL) is an aggressive leukemia which comprises 15% and 25% of pediatric and adult ALL, respectively. T-ALL is generally associated with poor prognosis and affected patients are at a high risk of remission induction failure and early relapse. Despite modern chemotherapy protocols and intensive regimens of multi-agent chemotherapy, the outcome of patients with refractory/relapsed disease did not improve significantly, which further highlights the necessity of combination therapies and new therapeutic approaches for this group of patients (1-3). 

Since 2003, when the first proteasome inhibitor (PI), bortezomib, received approval for clinical application, not only the treatment of multiple myeloma has revolutionized but also the management approaches to other malignancies, including mantle cell lymphoma (MCL), acute myeloid leukemia (AML), acute lymphoblastic leukemia (ALL) and even some solid tumors has been changed (4). Currently, proteasome inhibition is regarded as an attractive therapeutic strategy in treatment of refractory/relapsed ALL patients and several studies have been devoted to explore the efficacy and clinical benefits of PIs on these cases. It has been indicated that bortezomib and the second generation of proteasome inhibitor carfilzomib (Cfz), can overcome resistance to specific chemotherapeutic drugs (5, 6). Junk *et al.* showed that bortezomib combination therapy with prednisolone can enhance the cytotoxic effects of prednisolone on pediatric B cell precursor ALL cell lines (7). The combination of bortezomib with the chemotherapy drugs was also reported to be an effective approach for pediatric patients with relapsed/refractory ALL, with the response rate of 71% in children with T-ALL (8). A phase I clinical trial of carfilzomib was also conducted by Wartman *et al. *for relapsed/refractory AML and ALL which indicated that this drug is safe and well-tolerated (9). 

Ubiquitin-proteasome system (UPS) has an integral role in protein degradation, which in turn regulates cellular processes, such as cell cycle, signal transduction, transcription and programmed cell death via proteolysis of their regulatory components. Proteasome degradation is not the only mechanism of cellular catabolism and autophagy is also another intracellular degradation system for maintaining cellular homeostasis. Autophagy is contributed to the degradation of long half-life proteins and cytoplasmic organelles through lysosomal digestion, while short half-life proteins are mainly targeted by UPS. Although it has been traditionally assumed that these two pathways are distinct from each other, today it seems that there is some crosstalk. It has been revealed that inhibition of UPS results in the increased autophagic flux as a compensatory mechanism (10). One of the mechanisms which is probably involved in the interplay of UPS and autophagy is the unfolded protein response (UPR) (10). The accumulation of misfolded or unfolded proteins in endoplasmic reticulum (ER) results in the ER stress, which in turn activates the UPR (11). Primarily, UPR serves as a protective mechanism but in sustained stress conditions, such as presence of PIs, it directs the cells toward programmed cell death (12). 

The present study aimed to assess the effects of the proteasome inhibition by carfilzomib on the induction of apoptosis and autophagy in Molt4 cells. The effect of carfilzomib in combination with dexamethasone (Dex) in Molt4 cells was also investigated. 

## Experimental

*Cell line and drugs*

Molt4 cells, a human T-ALL derived cell line, were grown in RPMI-1640 medium supplemented with 10% heat-inactivated fetal bovine serum, 100 U/mL penicillin and 100 µg/mL streptomycin (PAN Biotech, Germany). The cells were cultured in a humidified atmosphere with 5% CO_2_ at 37 ºC. Stock solutions of Dex (Sigma-Aldrich) and Cfz (Sigma-Aldrich) were prepared by dissolving the compound in 0.1% dimethyl sulfoxide (DMSO, Sigma-Aldrich), which then were further diluted in fresh RPMI-1640 medium in desired concentrations.

*Trypan blue exclusion assay*

The effect of Cfz on cell viability of Molt4 cells was explored using trypan blue dye exclusion assay. Cells (at a density of 3 × 10^5 ^cells per mL) were seeded in a 24-well microplate and treated with various concentrations of Cfz. After 24 h and 48 h incubation, one part of the cell suspension was mixed with one part of 0.4% trypan blue solution (1:1 ratio). The mixture was incubated in room temperature for 1-2 min and then loaded onto the chamber of a Neubauer hemocytometer. The percentage of viable cells was then calculated by dividing viable cell count to total cell count and multiplying the result to 100.

*Microculture tetrazolium test (MTT)*

For investigating the metabolic activity of treated cells, microculture tetrazolium test (MTT) was performed. For this purpose, cells (at a density of 3 × 10^4^ cells per well) were seeded in 96-well flat-bottomed microplate and incubated with increasing concentrations of drugs for 24 h and 48 h. Then, the cells were incubated with 10 μL MTT solution [5 mg/mL in phosphate-buffered saline (PBS)] for 4h at 37 ºC. In the next step, the medium was removed and established formazan crystals were solubilized by adding 100 μL DMSO to each well. Finally, the absorbance of each well was measured at the wavelength of 570 nm in an ELISA microplate reader. 

*Cell apoptosis assay using Annexin V-FITC /PI double staining*

The effect of Dex and Cfz on cell death of Molt4 cells was explored by flow cytometry. Briefly, cells were seeded onto 6-well cell culture plate at a density of 1 × 10^6^ cells/well. Following 48 h treatment with drugs, cells were collected and washed with cold PBS and were resuspended in 100 μL reaction buffer containing 5 μL Annexin V–FITC and 5 μL PI. Cell solutions were then incubated for 15 min at room temperature in the dark. Finally, 400 μL of the binding buffer was added to each sample and then the fluorescence was quantified by flow cytometry. Positive staining for Annexin V is indicative of apoptosis and PI staining differentiates early and late apoptotic phases; as cells with positive Annexin V and negative PI are in early apoptotic phase, while cells which are positive for both Annexin V and PI underwent late apoptosis. 

*Flow cytometric analysis of cell cycle*

Cell cycle progression was assessed using propidium iodide (PI) staining following 48 h incubation of cells (1 × 10^6 ^cells per sample) with various concentrations of Cfz. Thereafter, the cells were collected and washed with PBS. After fixation with 70% ethanol, the cells were treated with PI and RNase at 37 ºC for half an hour. DNA content of the cells was quantified from the peak analysis of flow cytometric histograms, and the data were interpreted using the FlowJo 7.6.1 software.

*Detection of intracellular reactive oxygen species*

Generation of intracellular reactive oxygen species (ROS) mediated by Cfz was investigated using DCFH-DA staining. It is a fluorogenic dye which diffused into the cells and cleaved by nonspecific esterases, a reaction which converts DCFDA to DCFH. DCFH is then oxidized by ROS into the fluorescent compound DCF which can be detected by flow cytometery. Molt4 cells were treated with Cfz and following incubation time, the cells were incubated with DCFH-DA at 37 ºC for 30 min. Then the fluorescence of each sample was quantified by flow cytometry.

*RNA extraction and reverse transcription*

Total RNA was extracted manually from 1 × 10^6^ cells using RiboEx^TM^ Total RNA isolation solution (GeneAll, Korea). The quantity of extracted RNA was evaluated by NanoDrop spectrophotometer. Complementary DNA (cDNA) was prepared by using PrimeScript^TM^ RT Reagent Kit (Takara Bio Inc., Japan) according to the manufacturer’s instructions. 

*Quantitative real-time reverse transcriptase -PCR*

The cDNA templates were subjected to quantitative real-time reverse transcriptase-PCR by means of the Rotor-Gene Q real time RT-PCR system (Qiagen, Velencia, CA) using specific gene primers and SYBR Green PCR Master Mix (Ampliqon, Odense, Denmark). Fold-change values in gene expression were calculated using 2^−ΔΔCT^ formula. The quantity of total RNA in each sample was normalized by using ABL as an endogenous reference gene. Untreated cells were considered as control. The primer sequences are provided in Table 1.

*Detection of autophagy by acridine orange staining*

Molt4 cells were treated with increasing concentrations of Cfz for 48 h and washed with PBS for three times. The cells were then stained with 1 μg/mL acridine orange (Merck, Darmstadt, Germany) for 15 min at 37 ºC while protecting from light. Finally, a fluorescence microscope was used for visualizing the cells. Acridine orange is a green fluorophore which can cross and accumulate in acidic vesicular organelles such as autophagic lysosomes and generates an orange/bright red fluorescent, while other cellular compartments remain green. 

*Determination of combined drug effect*

The interaction between Cfz and Dex was investigated by calculating the combination index (CI) and the dose reduction index (DRI) using CompuSyn software and the method of Chou and Talalay (13). According to the classic isobologram equation, the CI and DRI values were calculated as follows: CI = (D)_1_/(Dx)_1_ + (D)_2_/(Dx)_2_, DRI_1_ = (Dx)_1_/(D)_1_ and DRI_2_ = (Dx)_2_/(D)_2_. (Dx)_1_ and (Dx)_2_ are the individual dose of Dex and Cfz which is required for a given level of effect and D_1_ and D_2_ are doses of Dex and Cfz which are required for producing the same effect in combination, respectively. The CI values of <1 are defined as synergistic effect and values =1 as additive, while values of >1 are considered antagonistic. 

*Statistical analysis*

Data are indicated as mean ± standard deviation (SD) of three independent experiments. For comparing groups paired sample t-test or one-way ONOVA and Dunnet post-hoc test were performed. *P*-values of less than 0.05 were considered as statistically significant.

## Results

*Cfz induced cytotoxic effects on Molt4 cells*

**Figure 1:**
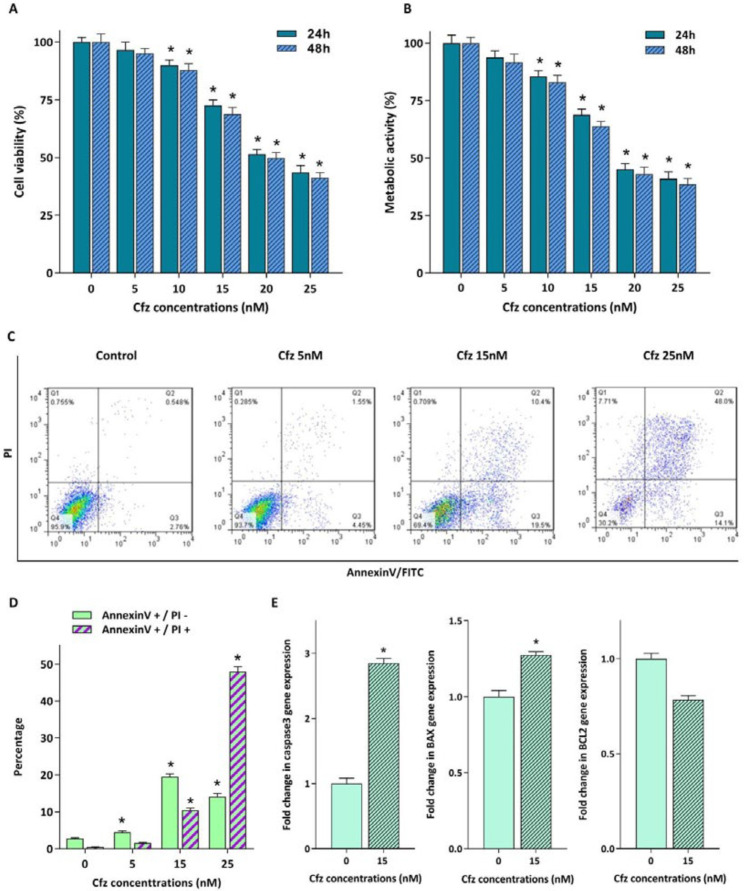
Cytotoxic effect of increasing concentrations of Cfz on Molt4 cells. (A and B) Exposure of the cells to the different concentrations of the agent reduced both the viability and the metabolic activity of the cells. (C) Flow cytometric analysis of the Cfz-induced apoptosis in Molt4 cells following 48 h of treatment using Annexin V-FITC/PI double staining. (D) Quantified analysis of the early (AnnexinV +/PI – cells) and late (AnnexinV +/PI + cells) apoptotic phases. (E) The mRNA expression levels of caspase3, Bax and Bcl2 in Molt4 cells treated with 15 nM Cfz compared with untreated cells as control. Values are shown as mean ± SD of three independent experiments. *P*-values ≤ 0.05 are indicated by asterisk and considered as statistically significant changes compared with control

For evaluating the cytotoxic effects of proteasome inhibition on Molt4 cells, the cells were treated with increasing concentrations of Cfz (5, 10, 15, 20 and 25 nM) up to 48 h. The results of trypan blue staining and MTT assay indicated that both the viability and the metabolic activity of the cells decreased in response to drug treatment (Figures 1A and 1B). To confirm the cytotoxic effect of Cfz, we performed Annexin V-FITC/propidium iodide (PI) double staining assay. As shown in Figures 1C and 1D, a significant increase in the proportion of apoptotic cells was observed in a dose-dependent manner. The results of qRT-PCR analysis also showed that while the mRNA expression levels of caspase3 and Bax were elevated in response to Cfz treatment, the expression level of anti-apoptotic gene Bcl2 decreased (Figure 1E).

*Cfz arrested cells in the G2/M phase of the cell cycle*

**Figure 2:**
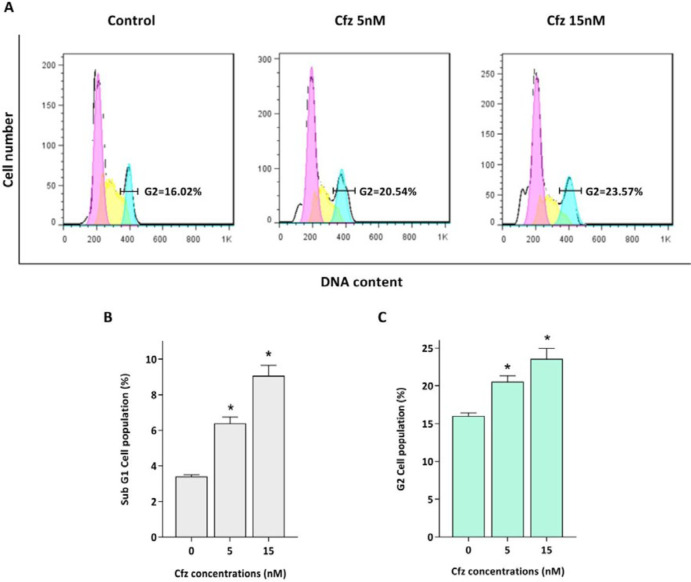
The effect of Cfz on cell cycle progression after 48 h of treatment. (A) Cfz increased the number of the cells in the G2/M phase of the cell cycle. (B) Quantitative analysis of the results showed that proteasome inhibition increased the population of Molt-4 cells in the sub-G1 phase. (C) Cfz induced G2/M arrest. *P*-values ≤ 0.05 are indicated by asterisk and considered as statistically significant changes compared with control

Proteasomal degradation serves as an integral regulator for cell cycle progression through proteolysis of the main components of the cell cycle machinery including cyclins and cyclin dependent kinas (CDK) inhibitors. Given this, the effect of Cfz on the cell distribution in the cell cycle was evaluated. As depicted in Figure 2, PI staining of treated cells showed that the population of the cells increased in sub-G1 phase of the cell cycle, which is a marker for apoptosis induction. The results also showed that Cfz induced G2/M arrest in Molt4 cells. 

*Cfz triggered ROS generation was accompanied by the increase in the expression of FOXO3a and SIRT1*

**Figure 3:**
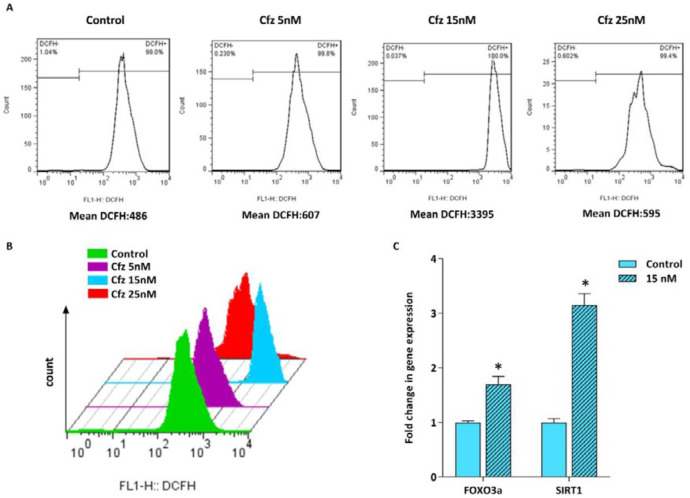
Flow cytometric analysis of ROS generation in Molt4 cells following treatment with increasing concentrations of Cfz. (A and B) Cfz increased the production of intracellular ROS in Molt4 cells. (C) The mRNA levels of FOXO3a and SIRT1 in Molt4 cells treated with 15 nM Cfz as compared with the untreated cells. Values are shown as mean ± SD of three independent experiments. *P*-values ≤ 0.05 are indicated by asterisk and considered as statistically significant changes compared with control

It has been suggested that proteasome inhibition resulted in accumulation of the unfolded and misfolded proteins in cancer cells leading to elevation of the cellular stress (11, 14). Given this, we assumed that cfz-induced apoptosis might be mediated through ROS production. Therefore, the effect of Cfz on ROS generation in Molt4 cells was assessed by flow cytometry using DCFH staining. As represented in Figures 3A and 3B, ROS production was dramatically induced with 15 nM Cfz. However, when we elevated the concentrations of Cfz to more than its IC_50_ value, the amount of ROS level reduced in the cells probably due to the increased number of apoptotic cells. For further investigation of oxidative stress, we also assessed the expression level of FOXO3a and SIRT1 genes which are two critical regulators of oxidative stress. The results of q-RT PCR showed that both the mRNA level of FOXO3a and SIRT1 increased in response to the drug treatment. 

*Cfz induced autophagy in Molt4 cells was coupled with the increased expression of CHOP*

**Figure 4:**
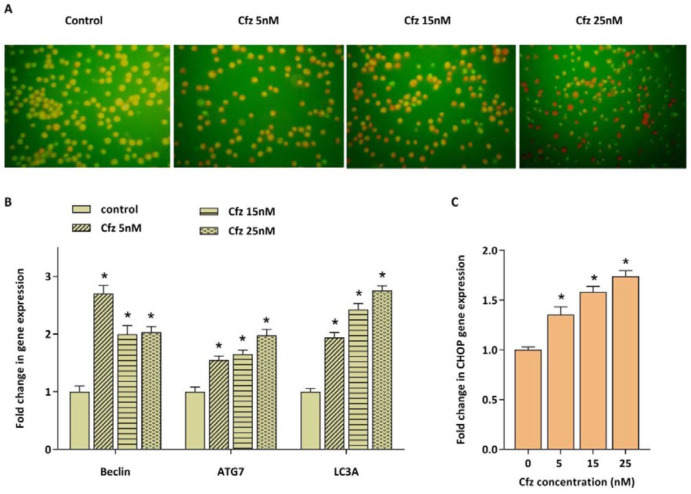
The effect of Cfz on the induction of autophagy in Molt4 cells after 48 h of treatment. (A) Acridine orange staining of Molt4 cells after treatment with Cfz (5, 15 and 25 nM) was suggestive of the induction of autophagy. (B and C) The mRNA expression levels of beclin1, ATG7, LC3A and CHOP in Cfz-treated Molt4 cells. Values are shown as mean ± SD of three independent experiments. *P*-values ≤ 0.05 are indicated by asterisk and considered as statistically significant changes compared with control

Accumulation of unfolded and misfolded proteins as a consequence of inhibition of proteasomal degradation also results in the enhancement of autophagy flux as a compensatory mechanism. The effect of Cfz on autophagy induction in Molt4 cells was evaluated by acridine orange staining. We also evaluated the mRNA expression levels of three main autophagy related genes including beclin1, ATG7 and LC3A. As presented in Figures 4A and 4B, Cfz induced autophagy in Molt4 cells. To investigate whether ER stress and UPR are associated with the autophagy induction, the mRNA expression level of CHOP was further evaluated. CHOP is considered as a critical target gene of ATF4 which its expression increases following activation of the UPR pathway and serves as a key transcription factor for promoting the expression level of several pro-apoptotic and autophagic genes. The data obtained by q-RT PCR demonstrated the enhancement in the expression of CHOP upon exposing Molt4 cells to Cfz (Figure 4C). 

*Cfz synergized the cytotoxic effect of Dex on Molt4 cells*

**Figure 5:**
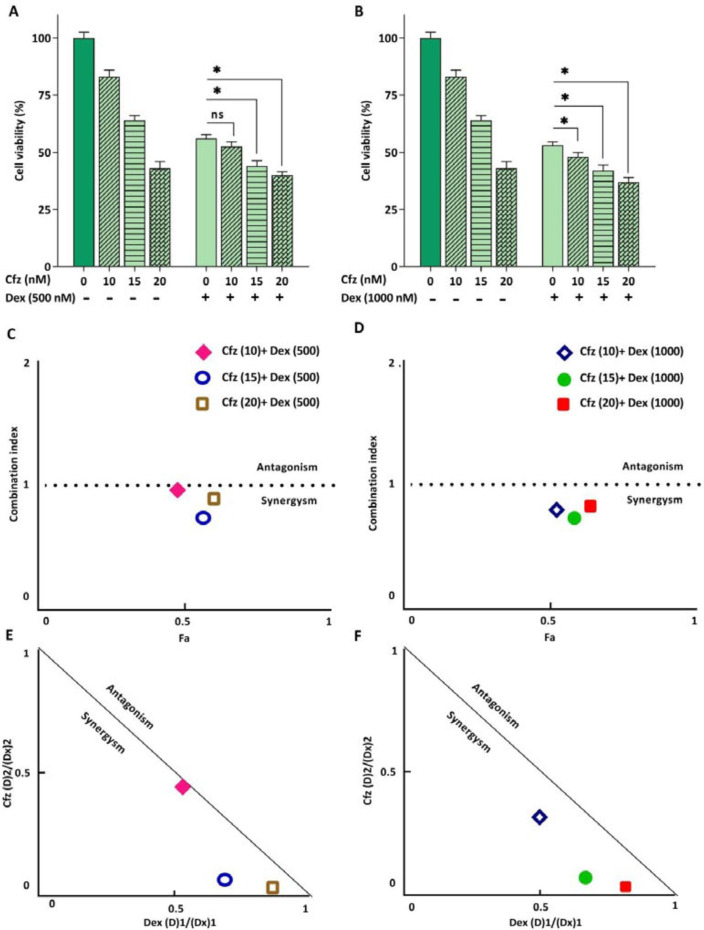
(A and B) The effect of Cfz in combination with Dex on the metabolic activity of Molt4 cells after 48 h of treatment. (C and D) Combination index (CI) analysis of Dex and Cfz (E and F) and the normalized isobolograms. Values are shown as mean ± SD of three independent experiments. ns: not significant

**Figure 6:**
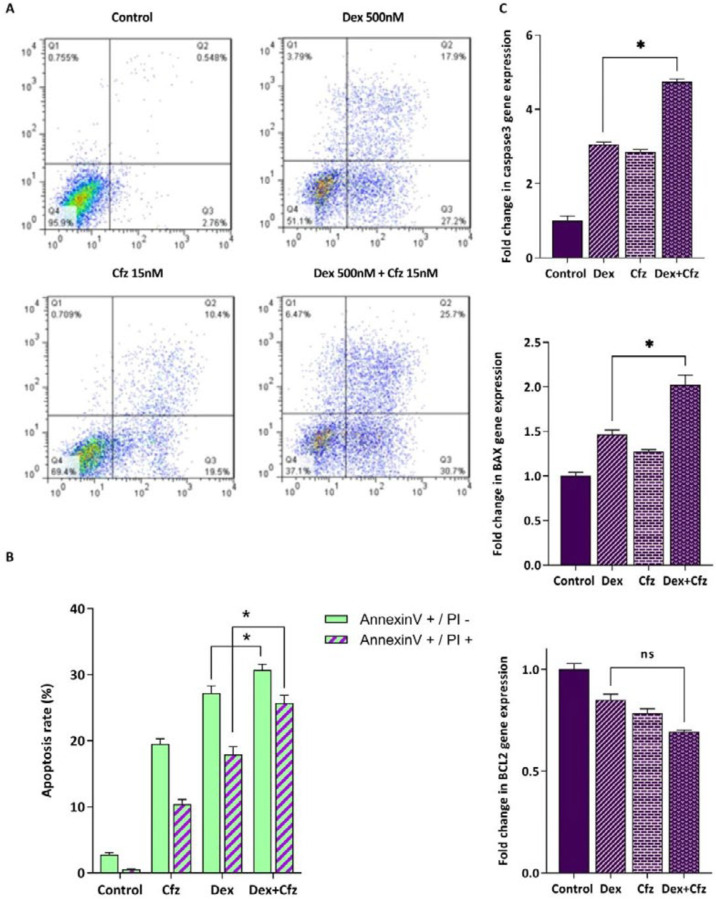
(A) Flow cytometric analysis of the apoptosis induced by Cfz (15 nM) and Dex (500 nM) in Molt4 cells after 48 h of treatment using Annexin V-FITC/PI double staining. (B) Quantified analysis of the early (AnnexinV +/PI – cells) and late (AnnexinV +/PI + cells) apoptotic phases. (C) The mRNA expression levels of caspase3, Bax and Bcl2 in Molt4 cells after treatment with both Dex (500 nM) and Cfz (15 nM) as compared with single drug treatment. Values are shown as mean ± SD of three independent experiments. *P*-values ≤ 0.05 are indicated by asterisk and considered as statistically significant changes compared with control. ns: not significant.

Glucocorticoids (GCs) are one the main drugs used in ALL treatment protocols, and the patient’s response to initial GC therapy is regarded as an indicative factor which may predict the overall outcome of treatment. Considering the key role of GCs in treatment of ALL patients, it was of great interest to investigate the interaction between Cfz and Dex on Molt4, a model of GC-resistant T-ALL cells. As indicated in Figure 5, the results obtained by CompuSyn software indicated that the effect of Cfz in combination with Dex was synergistic (CI values < 1). Combination index (CI) and dose reduction index (DRI) for drug combinations are provided in Table 2. The combination of Cfz at the concentration of 15 nM and Dex at the concentration of 500 nM was selected for further investigations (Figures 6A and 6B). As demonstrated in Figure 6C, there was a statistically significant difference in the expression levels of the caspase3 and Bax when Cfz was used in combination with Dex, as compared to either drug alone. 

*Increased apoptosis rate of Molt4 cells in concomitant treatment of Cfz and Dex is coupled with the enhancement of autophagy*

**Figure 7:**
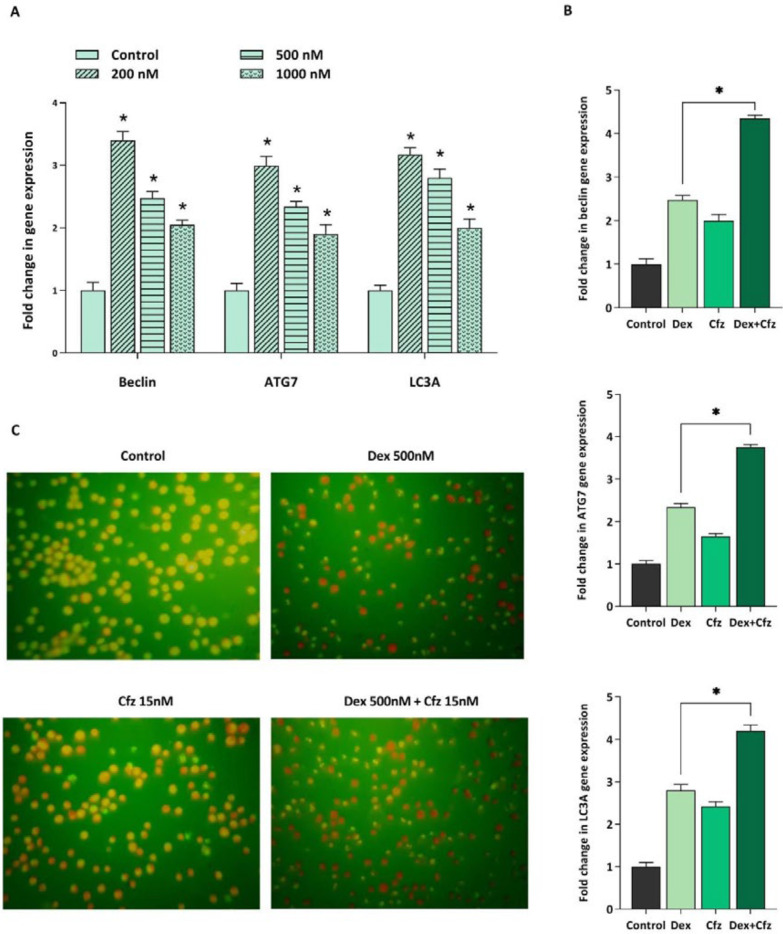
(A) The mRNA expression levels of beclin1, ATG7 and LC3A in Molt4 cells after 48 h of treatment with Dex alone (200, 500 and 1000 nM), (B) and in concomitant treatment of Dex- (500 nM) plus-Cfz (15 nM). (C) Acridine orange staining of Molt4 cells after treatment with Cfz (15 nM) and Dex (500 nM) is suggestive of the involvement of autophagy in the drugs-induced cytotoxic effects. Values are shown as mean ± SD of three independent experiments. *P*-values ≤ 0.05 are indicated by asterisk and considered as statistically significant changes compared with control

It was previously reported that for overcoming GC resistance in pediatric ALL cell lines, it is necessary to induce autophagy. Accordingly, it was of particular interest to investigate whether autophagy induction is contributed to the increased cytotoxicity of Dex in concomitant treatment of Cfz. For this purpose, we assessed the effect of Dex, alone and in combination with Cfz, on the autophagy flux in Molt4 cells. As indicated in Figure 7A, the mRNA expression levels of autophagy-related genes, including beclin1, ATG7 and LC3A were significantly higher in Dex-treated cells than in untreated cells. Our results also represented that combined treatment of Molt4 cells with Dex and Cfz resulted in a more escalation in the expression levels of autophagy related genes. Therefore, it seems that the enhanced cytotoxic effects of Dex on Molt4 cells in combination with Cfz, may be due to the induction of autophagic flux in this cell line.

## Discussion

The implication of UPS in protein degradation which in turn regulates cellular processes such as cell cycle, signal transduction, transcription and programmed cell death, makes this system an attractive target for cancer therapy. The application of proteasome inhibitors in combination treatments for relapsed/refractory ALL patients has been progressively increased in the last decade. Although there are several studies exploring the efficacy of PIs on pre B ALL (7, 15-17), the data about both the efficacy and the underlying mechanisms of PI action are still limited in T-ALL. Our data showed that Cfz, can induce both apoptosis and autophagy in Molt4 cells which is coupled with the increased CHOP expression. CHOP is a critical pro-apoptotic component of the UPR pathway which acts as a downstream target of PERK and ATF4. It serves as a transcription factor, alone or in combination with ATF4, and promotes transcription of several pro-apoptotic and autophagy-related genes. Esther *et al.* demonstrated that proteasome inhibitors enhanced the expression of pro-apoptotic component of the UPR including PERK, ATF4 and CHOP in multiple myeloma cell lines (18). It has been well-established that ER stress and UPR pathway are potent inducers of autophagy. According to a study by Kawaguchi *et al.* bortezomib induced both autophagy and ER stress in multiple myeloma cells (19). 

We also demonstrated that Cfz is a potent inducer of ROS production which in turn triggers double strand DNA breaks and thereby leads to the cell cycle arrest or apoptosis. Consistently, ROS generation is associated with the elevation in the expression of FOXO3a and SIRT1. SIRT1, an NAD^+^-dependent deacetylase, is regarded as a negative regulator of ER stress and according to the study of Koga *et al.*, ER stress leads to the upregulation of SIRT1 expression (20). Another study by Ghosh* et al. *showed that SIRT1 deficiency results in a delayed and suppressed expression of CHOP and GADD34 (21). The contribution of SIRT1 in regulation of CHOP and GADD34 further highlights the role of SIRT1 in the stress response pathway. It has been demonstrated that SIRT1 is also a positive regulator of autophagy via deacetylation of several autophagy-related genes. The critical role of SIRT1 in the oxidative stress-induced autophagy and apoptosis in mesenchymal- and hematopoietic- embryonic stem cells has been demonstrated by Ou *et al.* (22). FOXO3a is also another regulator of oxidative stress which can be activated by ROS. Furthermore, in stress conditions FOXO3a is deacetylated by SIRT1 and protects the cells from oxidative stress (23). FOXO3a can also induce autophagy through transcriptional upregulation of autophagy-related genes or autophagy regulatory genes. It has been demonstrated that FOXO3 activates PI3K/Akt signaling pathway which in turn leads to the translocation of FOXO1 to the cytoplasm that is necessary for FOXO3-induced autophagy (24). 

We also assessed the effect of Cfz on cell distribution of the cell cycle in Molt4 cells and found that this agent can induce G2/M cell cycle arrest. According to the literature, induction of G2/M arrest by PIs is mainly through activation of p53 and p21 (25). However, considering that Molt4 possesses mutant-p53, it is assumed that Cfz-induced G2/M arrest in these cells is mediated through p53-independent manner. 

Our data also showed that Cfz can sensitize Molt4 cells to Dex-induced apoptosis through a synergistic effect. Furthermore, as it was previously reported, we indicated that single treatment of cells with Dex was also coupled with the induction of autophagy. Laane *et al.* demonstrated that Dex can induce autophagy in ALL cells and it can initiate apoptosis (26). Furthermore, we found that concomitant treatment of Molt4 cells with Cfz and Dex enhanced the induction of autophagy as compared with each drug alone. Therefore, it seems that the increased cytotoxic effects of Dex on Molt4 cells in combination with Cfz, may be at least in some part, due to the enhancement of autophagic flux in this cell line. Consistently, Bonapace *et al.* revealed that the induction of autophagy is necessary for overcoming GC resistance in pre-B ALL cell lines (16). 

In conclusion, we found that proteasome inhibition by Cfz triggers both apoptosis and autophagy in Molt4 cells which are partly due to the induction of ER stress. Furthermore, we showed that Cfz can enhance the cytotoxicity of Dex. These findings further indicated the underlying mechanisms of proteasome inhibition in a T-ALL cell line and may be suggestive of the promising role of PIs in combination therapies for relapsed/refractory T-ALL patients.

**Table 1:** The primer sequences used for real-time PCR

**Gene**	**Forward primer (5’-3’)**	**Reverse primer (5’-3’)**	**Product size (bp)**
Abl	CTTCTTGGTGCGTGAGAGTGAG	GACGTAGAGCTTGCCATCAGAAG	115
Bax	GATGCGTCCACCAAGAAGCT	CGGCCCCAGTTGAAGTTG	170
Bcl2	ATGTGTGTGGAGAGCGTCAACC	TGAGCAGAGTCTTCAGAGACAGCC	196
Caspase3	AAATACCAGTGGAGGCCGACT	TCAGCATGGCACAAAGCGAC	114
Beclin	TGCAGGTGAGCTTCGTGTG	CTGGGCTGTGGTAAGTAATGGAG	119
Atg7	ATTGCTGCATCAAGAAACCC	GATGGAGAGCTCCTCAGCA	121
LC3A	CGTCCTGGACAAGACCAAGT	CTCGTCTTTCTCCTGCTCGT	181
FOXO3a	CACGCACCAATTCTAACG	ACGGCTTGCTTACTGAAG	153
SIRT1	TCGCAACTATACCCAGAACATAGACA	CTGTTGCAAAGGAACCATGACA	86
CHOP	TTAAAGATGAGCGGGTGGCAG	CAGGTGTGGTGATGTATGAAGAT	101

**Table 2:** Combination index (CI) and dose reduction index (DRI) for drug combinations by carfilzomib and dexamethasone

**Carfilzomib**		**Dexamethasone**		
**Concentration (nM)**	**DRI**	**Concentration (nM)**	**DRI**	**CI**
10	1.860	500	2.243	0.983
15	1.439	500	15.841	0.758
20	1.160	500	40.595	0.887
10	2.012	1000	3.147	0.814
15	1.492	1000	12.640	0.749
20	1.226	1000	41.951	0.840
